# Maggots, Mucous and Monkey Meat: Does Disgust Sensitivity Affect Case Mix Seen During Residency?

**DOI:** 10.5811/westjem.2019.9.44309

**Published:** 2019-12-09

**Authors:** Benjamin H. Schnapp, Emily Fleming, Aaron S. Kraut, Mary Westergaard, Robert J. Batt, Brian W. Patterson

**Affiliations:** * University of Wisconsin School of Medicine and Public Health, BerbeeWalsh Department of Emergency Medicine, Madison, Wisconsin; † University of Wisconsin, Department of Operations and Information Management, Madison, Wisconsin

## Abstract

**Introduction:**

Emergency physicians encounter scenarios daily that many would consider “disgusting,” including exposure to blood, pus, and stool. Physicians in procedural specialties such as surgery and emergency medicine (EM) have lower disgust sensitivity overall, but the role this plays in clinical practice is unclear. The objective of this study was to determine whether emergency physicians with higher disgust sensitivity see fewer “disgusting” cases during training.

**Methods:**

All EM residents at a midsize urban EM program were eligible to complete the Disgust Scale Revised (DS-R). We preidentified cases as “disgust elicitors” based on diagnoses likely to induce disgust due to physician exposure to bodily fluids, anogenital anatomy, or gross deformity. The “disgust elicitor” case percent was determined by “disgust elicitor” cases seen as the primary resident divided by the number of cases seen thus far in residency. We calculated Pearson’s r, t-tests and descriptive statistics on resident and population DS-R scores and “disgust elicitor” cases per month.

**Results:**

Mean DS-R for EM residents (n = 40) was 1.20 (standard deviation [SD] 1.24), significantly less than the population mean of 1.67 (SD 0.61, p<0.05). There was no correlation (r = −0.04) between “disgust elicitor” case (n = 2191) percent and DS-R scores. There was no significant difference between DS-R scores for junior residents (31.1, 95% confidence interval [CI], 26.8–35.4) and for senior residents (29.0, 95%CI, 23.4–34.6).

**Conclusion:**

Higher disgust sensitivity does not appear to be correlated with a lower percentage of “disgust elicitor” cases seen during EM residency.

## INTRODUCTION

Selecting a specialty is one of the most impactful choices a physician makes in his or her career, affecting lifestyle, salary, and happiness.^1^ Medical students are advised to consider the value they place on patient contact, longitudinal care, research, and procedural skill.^2^ More recent evidence suggests that personality may also play a role in specialty choice.^3^,^4^ Recently, the emergency medicine (EM) personality was found to be markedly different from that of other physicians: emergency physicians tend to be more vigilant, team-oriented, and pragmatic.^5^

Disgust is an emotion thought to have evolved to encourage humans to avoid disease transmission;^7^ however, there are significant individual differences in disgust sensitivity.^8^ To successfully care for patients afflicted with infections, vomiting, or anogenital issues among others, physicians must manage their innate disgust response by donning gloves, masks and gowns, or simply by accepting the necessity of the exposure in the name of patient care. Studies have previously shown that lower disgust sensitivity correlated with a choice of nursing or medicine over pharmacology.^9^ However, not all medical specialties require equal exposure to “disgust elicitors,” and prior research has shown lower disgust sensitivity in those planning to choose a procedural specialty such as surgery or EM.^10^

While emergency physicians may have lower disgust sensitivity overall, it is not known whether individual differences in disgust sensitivity impact clinical performance during residency. EM residents have previously been shown to “cherry-pick” the patient cases that they see;^11^ if residents with higher disgust sensitivity select fewer “disgust elicitor” cases, they could leave training with skill and knowledge gaps compared to their less sensitive peers. Additionally, medical students considering EM may find it valuable to know whether their propensity for disgust could affect their future career success. The purpose of this study was to determine whether emergency physicians with higher disgust sensitivity see fewer “disgusting” cases during training.

## METHODS

This was a cross-sectional retrospective study conducted at a three-year academic, midsize city residency program in the midwest with 12 residents per year. Residents from graduation years (GY) 2018 to 2021 were eligible. Participants were asked to complete the Disgust Scale Revised (DS-R), a 25-item, validated, disgust sensitivity scale^12^,^13^ that has been shown to have behavioral correlates (Appendix A).^14^ Participants were informed that their results would be confidential, used for investigation only, and not used as part of any evaluation for residency.

We extracted the top 1000 ICD-10 billing codes for the last four years from the electronic health record (EHR). Sixty-two “disgust elicitors” were chosen from this list by a consensus group of three experienced emergency physicians based on likely physician exposure to phenomena generally regarded by the public as “disgusting,” including bodily fluids, anogenital anatomy, or gross physical deformity (Appendix B). Borderline examples such as “vomiting” were excluded as the physician was not guaranteed to be exposed directly to the disgusting attribute. Similarly, broad diagnoses such as “infected lower extremities” were excluded as these were felt to represent too wide a variation in clinical presentation, from the disgust-eliciting purulent wound to the minimally bothersome early leg cellulitis.

For each resident, the “disgust elicitor” case percent was determined by querying the EHR for “disgust elicitor” cases seen as the first assigned resident over the entire course of his or her residency thus far, and then dividing by the total number of cases seen as the first assigned resident at the residency’s main emergency department (ED) site. Taking over care of a patient with a “disgust elicitor” diagnosis was not counted toward a resident’s total, as it was felt that the “disgust elicitor” aspect (e.g., rectal exam) was likely addressed by the first physician. We calculated a Pearson’s r between resident DS-R scores and their “disgust elicitor” case percent; descriptive statistics and t-tests were calculated on resident and population DS-R scores.^15^

This study was determined to be exempt by the University of Wisconsin IRB.

## RESULTS

Of 48 eligible residents, 42 (87.5%) completed the DS-R. One response was removed from the analysis per the DS-R scoring recommendations for indicating a high level of disgust to a distractor question; another was removed as the respondent could not be matched to cases. Ultimately we analyzed data from 40 residents, representing 84,822 total cases. Median DS-R in the study population was 1.18; mean DS-R was 1.20 (standard deviation [SD] 1.24), significantly less (one sample t(39) = −4.8, p<0.01; Cohen’s d effect size = .7756, which can be interpreted as an intermediate effect^16^) than the population mean of 1.67 (SD 0.61). Individual disgust scores ranged from 0.36 to 2.28.

We identified 2191 total “disgust elicitor” cases that were seen primarily by study participants, representing 2.6% of the total cases. We found no correlation (r = −0.04) between “disgust elicitor” case percent and DS-R scores. See Table 1 for “disgust elicitor” cases broken down by each class. There was no significant difference (p = 0.56) between the mean DS-R scores for junior (graduation year [GY] 2020 and 2021) residents (31.1, 95% CI, 26.8–35.4) and for senior (GY2018 and GY2019) residents (29.0, 95% CI, 23.4–34.6).

## DISCUSSION

Our study suggests that greater disgust sensitivity does not correlate with a lower percentage of “disgust elicitor” cases seen by EM residents during their training. Consistent with prior research, disgust sensitivity was lower among EM residents compared with population means.^10^ Furthermore, disgust sensitivity was not significantly different between junior and senior residents.

There are several plausible explanations why there was no negative correlation between disgust sensitivity and “disgust elicitor” cases seen by EM residents. This could be due to an expectation that EM residents assign themselves to the next patient to be seen as determined by acuity or length of stay. Resident biases against “disgusting” chief complaints may be masked by the desire to conform to expectations of assigning oneself to the patient “next to be seen.” However, this idea is not supported by previous findings on EM resident “cherry picking.”^11^ Alternatively, physicians choosing EM may already meet a threshold for tolerance of “disgusting” cases that renders preference against individual patient presentations moot.

We did not see evidence in our study for lower overall disgust sensitivity for residents with additional years of training. A previous pilot study suggested that exposure could decrease disgust sensitivity,^17^ but it is possible that disgust sensitivity is more innate than malleable. Alternatively, residents’ disgust sensitivity could have been previously lowered by exposure during medical school, with floor effects preventing subsequent lowering during residency. The time course measured by this study may also have been too short to detect an effect if disgust sensitivity decreases over years of exposure to disgusting stimuli instead of weeks or months; this represents an avenue for future research.

While EM residents overall had a significantly lower disgust sensitivity than the general population, it is interesting to note that there was significant individual variation. Several residents, including two recent chief residents, had disgust sensitivity significantly higher than the population average. While this too represents an area for further study, it suggests that low disgust sensitivity is not a prerequisite for success in the field of EM. Future researchers may be interested to investigate the whether sustained exposure to “disgust elicitors” in residents with high disgust sensitivity has the potential to contribute to burnout.

## LIMITATIONS

Our study has several important limitations. This was a single-site study with a relatively small sample size. The DS-R results were confidential but not anonymous due to the need to match with cases, which may have affected how willing residents were to answer honestly. Although we attempted to choose cases that guaranteed residents were exposed to a “disgust elicitor,” as this was a retrospective chart review, cases were not individually probed to determine the extent of residents’ actual exposure to “disgusting” stimuli. The “disgust elicitor” cases selected also may have systematically missed relevant exposures; for example, tracheostomy problems or ophthalmologic complaints may induce significant disgust in certain clinical circumstances, or in certain individuals and not in others.

The cases identified for this study were from the EHR system used at the main hospital site. Residents also rotate at several other clinical sites, including the Veteran’s Affairs hospital, an unaffiliated community site, and on electives at various global health sites. As such, we were unable to account for the complete range of clinical exposures during residency. The unpredictable nature of the ED clinical environment overall means that some residents may have had greater opportunities to see patients with “disgusting” complaints than others. Similarly, residents may have had other exposure to disgust elicitors prior to residency in careers such as nurse, ski patroller, or emergency medical technician. Other life experiences such as raising children or caring for older adults may have also exposed residents to disgust elicitors. Despite the difficulty of quantifying the nature of these experiences, it is possible that they may have exerted a global effect on our results.

## CONCLUSION

Our study confirms that EM resident physicians as a group have a lower disgust sensitivity compared with the general population. However, a higher individual disgust sensitivity does not correlate with a lower percentage of “disgust elicitor” cases seen. Medical students who are considering EM but are wary because of their sensitivity to “disgust elicitors” may be reassured that low disgust sensitivity does not appear to be required for success in EM.

## Supplementary Information





## Figures and Tables

**Table 1:** Total cases seen by each class, with number and percent of “disgust elicitor” cases.

	GY2021	GY2020	GY2019	GY2018
Total cases seen (95%CI)	434 (170–698)	1566 (1153–1978)	2839 (2127–3552)	3529 (2907–4152)
Number of “disgust elicitor” cases (95% CI)	13 (2–24)	43 (29–57)	71 (46–96)	89 (59–119)
Percent of “disgust elicitor” cases (95% CI)	3.0% (1.8–4.2)	2.8% (2.1–3.4)	2.5% (1.8–3.3)	2.5% (1.9–3.1)

*CI*, confidence interval; *GY*, graduation year.

## References

[1] Peckham C (2018). Medscape Physician Lifestyle & Happiness Report.

[2] Totman A (2015). Roadmap to Choosing a Medical Specialty.

[3] Hojat M, Zuckerman M (2008). Personality and specialty interest in medical students. Med Teach.

[4] Jafrani S, Zehra N, Zehra M (2017). Assessment of personality type and medical specialty choice among medical students from Karachi; using Myers-Briggs Type Indicator (MBTI) tool. J Pak Med Assoc.

[5] Jordan J, Linden JA, Maculatis MC (2018). Identifying the emergency medicine personality: a multisite exploratory pilot study. AEM Educ Train.

[6] Jaime JA, LJ CMM (2018). Identifying the emergency medicine personality: a multisite exploratory pilot study. AEM Educ Train.

[7] Oaten M, Stevenson RJ, Case TI (2009). Disgust as a disease-avoidance mechanism. Psychol Bull.

[8] Inbar Y, Pizarro D, Iyer R (2012). Disgust sensitivity, political conservatism, and voting. Soc Psychol Pers Sci.

[9] Consedine NS, Yu TC, Windsor JA (2013). Nursing, pharmacy, or medicine? Disgust sensitivity predicts career interest among trainee health professionals. Adv Health Sci Educ Theory Pract.

[10] Consedine NS, Windsor JA (2014). Specific disgust sensitivities differentially predict interest in careers of varying procedural-intensity among medical students. Adv Health Sci Educ Theory Pract.

[11] Patterson BW, Batt RJ, Wilbanks MD (2016). Cherry picking patients: examining the interval between patient rooming and resident self-assignment. Acad Emerg Med.

[12] Olatunji BO, Williams NL, Tolin DF (2007). The Disgust Scale: item analysis, factor structure, and suggestions for refinement. Psychol Assess.

[13] Haidt J, McCauley C, Rozin P (1994). Individual differences in sensitivity to disgust: a scale sampling seven domains of disgust elicitors. Pers Individ Diff.

[14] Rozin P, Haidt J, McCauley C (1999). Individual differences in disgust sensitivity: Comparisons and evaluations of paper-and-pencil versus behavioral measures. J Res Pers.

[15] Haidt J (2010). Means for the DS-R.

[16] Cohen J (1988). Statistical power analysis for the behavioral sciences.

[17] Rozin P (2008). Hedonic “adaptation”: specific habituation to disgust/death elicitors as a result of dissecting a cadaver. Judgm Dec Mak.

