# Supplementary material for: Maggots, Mucous and Monkey Meat: Does Disgust Sensitivity Affect Case Mix Seen During Residency?
Source: West J Emerg Med. 2019 Dec 9;21(1):87–90. doi: 10.5811/westjem.2019.9.44309 (PMC6948699; doi:10.5811/westjem.2019.9.44309)
Supplement: Supplementary file 1 [file wjem-21-87-s001.docx]

**APPENDIX A** (The Disgust Scale-Revised, Haidt, McCauley & Rozin, 1994,^11^ modified by Olatunji et al. 2007^10^):

*Please indicate how much you agree with each of the following statements, or how true it is about you. Please write a number (0-4) to indicate your answer:*

**0** = Strongly disagree (very untrue about me)

**1** = Mildly disagree (somewhat untrue about me)

**2** = Neither agree nor disagree

**3** = Mildly agree (somewhat true about me)

**4** = Strongly agree (very true about me)

____1. I might be willing to try eating monkey meat, under some circumstances.

____2. It would bother me to be in a science class, and to see a human hand preserved in a jar.

____3. It bothers me to hear someone clear a throat full of mucous.

____4. I never let any part of my body touch the toilet seat in public restrooms.

____5. I would go out of my way to avoid walking through a graveyard.

____6. Seeing a cockroach in someone else's house doesn't bother me.

____7. It would bother me tremendously to touch a dead body.

____8. If I see someone vomit, it makes me sick to my stomach.

____9. I probably would not go to my favorite restaurant if I found out that the cook had a cold.

____10. It would not upset me at all to watch a person with a glass eye take the eye

out of the socket.

____11. It would bother me to see a rat run across my path in a park.

____12. I would rather eat a piece of fruit than a piece of paper

____13. Even if I was hungry, I would not drink a bowl of my favorite soup if it had been

stirred by a used but thoroughly washed flyswatter.

____14. It would bother me to sleep in a nice hotel room if I knew that a man had died of a

heart attack in that room the night before.

*How disgusting would you find each of the following experiences? Please write a*

*number (0-4) to indicate your answer:*

**0** = Not disgusting at all

**1** = Slightly disgusting

**2** = Moderately disgusting

**3** = Very disgusting

**4** = Extremely disgusting

____15. You see maggots on a piece of meat in an outdoor garbage pail.

____16. You see a person eating an apple with a knife and fork

____17. While you are walking through a tunnel under a railroad track, you smell urine.

____18. You take a sip of soda, and then realize that you drank from the glass that an

acquaintance of yours had been drinking from.

____19. Your friend's pet cat dies, and you have to pick up the dead body with your bare hands.

____20. You see someone put ketchup on vanilla ice cream, and eat it.

____21. You see a man with his intestines exposed after an accident.

____22. You discover that a friend of yours changes underwear only once a week.

____23. A friend offers you a piece of chocolate shaped like dog‑doo.

____24. You accidentally touch the ashes of a person who has been cremated.

____25. You are about to drink a glass of milk when you smell that it is spoiled.

____26. As part of a sex education class, you are required to inflate a new unlubricated

condom, using your mouth.

____27. You are walking barefoot on concrete, and you step on an earthworm.

To calculate your score: First, put an X through your responses to items 12 and 16 (these items don’t count). Then “reverse” your score on items 1,6, and 10 by subtracting what you wrote from the number 4, and write those numbers in the margin. Finally, add up your responses to all 25 items (using your “reversed” scores on 1, 6, and 10). The total will be a number between 0-100. For more information see: http://people.virginia.edu/~jdh6n/disgustscale.html
