# Supplementary material for: Maggots, Mucous and Monkey Meat: Does Disgust Sensitivity Affect Case Mix Seen During Residency?
Source: West J Emerg Med. 2019 Dec 9;21(1):87–90. doi: 10.5811/westjem.2019.9.44309 (PMC6948699; doi:10.5811/westjem.2019.9.44309)
Supplement: Supplementary file 2 [file wjem-21-87-s002.docx]

**APPENDIX B** *(List of Professional Billing Codes Selected by Group Consensus (of the top 1000 codes at the XXXX Emergency Department):*

Gastrointestinal hemorrhage, unspecified

Hemorrhage of anus and rectum

Abnormal uterine and vaginal bleeding, unspecified

Pelvic and perineal pain

Peritonsillar abscess

Melena

Acute vaginitis

Hematemesis

Rectal abscess

Other specified diseases of anus and rectum

Epidiymitis

Foreign body in nostril

Right testicular pain

Left testicular pain

Cutaneous abscess of buttock

Displaced trimalleolar fracture of right lower leg + left leg

Anal abscess

Unspecified ovarian cyst, left side

Female, pelvic inflammatory disease, unspecified

Other specified disorders of penis

Foreign body in urethra, initial encounter

Other specified abnormal uterine and vaginal bleeding

Complete or unspecified spontaneous abortion without complication

Residual hemorrhoidal skin tags

Threatened abortion

Epididymo-orchitis

Other specified disorders of the perineum

Other specified noninflammatory disorders of the vagina

Cutaneous abscess of abdominal wall

Retropharyngeal and parapharyngeal abscess

Cutaneous abscess of right lower limb

Anal fissure, unspecified

Inflammatory disorders of scrotum

Cutaneous abscess of neck

Rectal prolapse

Abscess of the breast and nipple

Torsion of testis, unspecified

Cutaneous abscess of left lower limb

Priapism, unspecified

Pilonidal cyst with abscess

Fecal impaction

Candidiasis of vulva and vagina

Abscess of vulva

Other hemorrhoids

Other specified disorders of male genital organs

Cellulitis of groin

Enterostomy malfunction

Foreign body in anus and rectum, initial encounter

Unspecified hemorrhoids

Cutaneous abscess of groin

Pilonidal cyst without abscess

Scabies

Cutaneous abscess, unspecified

Cellulitis of buttock

Testicular pain, unspecified

Other complications of colostomy

Foreign body in vulva and vagina, initial encounter

Cutaneous abscess of perineum

Scrotal pain

Unspecified ovarian cyst

Perianal venous thrombosis

Salpingitis and oophoritis
